# Naupathia

**Published:** 1888-07

**Authors:** 


### *NAUPATHIA.*

A paper was read before the French Academy of Sciences by M. Ossian-Bonnet, on the etiology and treatment of *naupathia* (seasickness). The author claims that the disease is purely of nervous origin; he is strongly inclined to discard the various mechanical theories that have been offered as explanation for it. He dismisses summarily succussion of the cerebral fluid, shock to the pneumogastric, movements of the viscera, etc. M. Ossian Bonnet regards vertigo as the characteristic symptom of naupathia; the nausea is the result only of digestive disturbances. He recalls the well-recognized fact that the motion of a ship often causes only vertigo. He mentions also the fact that some persons are affected only by the lateral motion of the ship, and others by the forward or pitching movement. He recalls to us that vertigo may be produced by the apparent instability of objects, known to be stable, as experience in swinging or on merry-go-rounds.

Assuming that the affection is purely "nervous," and that the nausea is the result of digestive derangement, M. Ossian-Bonnet arranged a rational method of treatment and took a sea trip from Havre to Buenos Ayres to test the correctness of his assumption.

A few of his suggestions are remarkably trite; he advises a saline laxative every morning for several days preceding the embarkment. If an attack of nausea comes on, he advises a glass of water to be drunk after each attack. He suggests that the latter measure ensures *lavage* of the stomach, and renders the vomiting less painful. Every one who has ever been "down" to the sea in a ship can endorse this little remarkable statement.

The writer, having observed the influence that antipyrine possesses in diminishing the excito-motor power of the spinal cord, concluded to try this drug in naupathia.

It is said that the remedy did not fail in a single case. It is gratifying to know that seasickness is no longer an inevitable ill for some, and that "sea-legs" may be got through antipyrine.

Among the many causes that have been given for seasickness, this is perhaps the most acceptable, seeing that, in using the word *nervous*, the author has left great room for speculation and pins us down to no succinct article of belief. Moreover, our therapeutics would be broad, for any agent that acted as a nervine would, *à priori*, be permissible in the treatment. Already a great variety of medicaments have been suggested for seasickness, and each one has been hailed as a possible specific. The bromides, morphine, atropine, and others saw their day, and went down finally to a rational insignificance. When cocaine was isolated and its local anæsthetic effect demonstrated, it was supposed that it possibly contained the balm for this troublesome ailment.

It was tried, and benefit was said to have been derived from it in many instances, but reason has also relegated it to its proper sphere.

The *British Medical Journal* recently contained a communication from Dr. J. J. Seiser, in which a mechanical explanation was suggested for seasickness, and a mechanical remedy was given. This observer had often remarked that there was an impulse to breathe irregularly on the part of any one affected by the motion of a ship. This disturbance of system in respiration would naturally cause temporary carbonic acid poisoning of the brain (presumably in the medulla). The symptoms of vertigo and nausea might be explained on this assumption.

The natural inference was, that if persons on ship-board could be got to breathe rhythmically, these two symptoms might be eliminated, by virtue of the elimination of the carbonic acid poisoning. This observer also took a sea trip, and put his theory to test. Forced rhythmical breathing was found to be very satisfactory as a treatment. This observation was corroborated by two other physicians who were associated with Dr. Seiser in the experiments.

No very long time has elapsed since some one suggested that seasickness was the result of an irritation seated in the semi-circular canals. This hypothesis took so firm a hold on the minds of those that promulgated and favored it, that they went about on ship-board, painting vesicating medicants on the mastoid region of unfortunates who were already subject to one of the most disagreeable ills that flesh is heir to. Whether this treatment was beneficial or not, it soon fell into disuse, for, probably, there was a question in the minds of the patients as to which was worse, blisters behind the ears, or naupathia. Possibly some had ultimately to endure both.

This theory is apparently rational, but the therapeutics which fit the theory perfectly are difficult to carry out, if not impracticable. Imagine a sea-sick person endeavoring to breathe at the rate of twenty respirations per minute. Their minds would certainly be employed and their attention directed away from their trouble—we doubt very much whether any one who is affected by seasickness would be able to breathe rythmically when he experiences the sensation as if the whole of creation were dropping away from his feet. Every one is familiar with the relief given by holding the breath in the descent of a swing. The desire to hold the breath in the downward swoop of a ship has often been noticed. If a man were to desist from breathing during an entire sea trip, he would probably not experience naupathia at all. Huxley has remarked that the "tendency of physico-chemical science is clearly towards the reduction of the problems of the world of the infinitely little, as it already has reduced those of the infinitely great world, to questions of mechanics."

The direct causes of all things are, perhaps, reducible to some form of mechanical force. Disease, like evil, is possibly the result of force misdirected, inadequate or excessive, for a given object; or a combination of the first with either of the latter two. This seems to us clearly to be the case in functional diseases.

Naupathia is certainly not an organic disease; it must be a functional one. There should, then, be a mechanical cause for it. All intelligent therapeutics are based upon the recognition of cause. The original cause of naupathia is certainly the movement of the ship. This movement disturbs the system in such a way that a series of symptoms and signs are produced. To prevent the movement, would be the first measure suggested by primitive reason. Such a thing is impracticable. The remaining therapeutic course is to prevent the secondary cause, which calls forth the symptoms of the affection. Whatever be the cause, it comes through the nervous system, and the symptoms are all expressed through it. To diminish the sensitiveness of the nervous system to the cause, is surely a rational measure. If an agent can be found to do this without deleterious or unpleasant after-effects, it should be given a fair trial.

The suggestion of antipyrine, by M. Ossian-Bonnet, is at least something new.

In most cases, one dose of twenty grains was sufficient, but in some it was necessary to repeat the dose or to give only in larger quantities. Let it be given a fair trial.

